# Magnetic record associated with tree ring density: Possible climate proxy

**DOI:** 10.1186/1467-4866-8-2

**Published:** 2007-03-24

**Authors:** Gunther Kletetschka, Petr Pruner, Daniela Venhodova, Jaroslav Kadlec

**Affiliations:** 1 Institute of Geology, AS CR, Prague, 16502, Czech Republic; 2 Department of Physics, Catholic University of America, Washington DC, 20064, USA; 3 Code 691, NASA's Goddard Space Flight Center, Greenbelt, MD, 20771, USA

## Abstract

A magnetic signature of tree rings was tested as a potential paleo-climatic indicator. We examined wood from sequoia tree, located in Mountain Home State Forest, California, whose tree ring record spans over the period 600 – 1700 A.D. We measured low and high-field magnetic susceptibility, the natural remanent magnetization (NRM), saturation isothermal remanent magnetization (SIRM), and stability against thermal and alternating field (AF) demagnetization. Magnetic investigation of the 200 mm long sequoia material suggests that magnetic efficiency of natural remanence may be a sensitive paleoclimate indicator because it is substantially higher (in average >1%) during the Medieval Warm Epoch (700–1300 A.D.) than during the Little Ice Age (1300–1850 A.D.) where it is <1%. Diamagnetic behavior has been noted to be prevalent in regions with higher tree ring density. The mineralogical nature of the remanence carrier was not directly detected but maghemite is suggested due to low coercivity and absence of Verwey transition. Tree ring density, along with the wood's magnetic remanence efficiency, records the Little Ice Age (LIA) well documented in Europe. Such a record suggests that the European LIA was a global phenomenon. Magnetic analysis of the thermal stability reveals the blocking temperatures near 200 degree C. This phenomenon suggests that the remanent component in this tree may be thermal in origin and was controlled by local thermal condition.

## Background

A cross section from coast redwood trees (*Sequoia sempervirens*) in Mountain Home State Forest, California, were dendrochronologically cross-dated (950–1450 years) and detected overall period between 600 and 1700 A.D. [1]. Tree ring density may detect climatic variations, however other factors, like fire frequency can also influence tree ring density [2]. Fire paleo-frequency can be detected by variability in formation of pedogenic magnetic particles [3] as well as by variability of paleo-climatic recorders [4]. Uptake of iron via roots requires incorporation of iron-rich solution from the soil and relies on absorption by the root system. Sapwood is the physiologically active part of the xylem (wood). This is the tissue through which water with dissolved iron moves from the roots to the shoots. The heartwood is the older, nonliving central wood of a tree that does not conduct water. Once the sapwood becomes hardwood, it is thermally isolated from the outside environmental changes. Up to three or four annual growth rings of xylem may be active in water transport. Because water movement is related to transpiration, environmental factors such as soil moisture, air temperature, and relative humidity affect the rate of water movement.

Sequoia species are long lived and contain cellular mechanism capable of slowing down or even stop telomere attrition [5]. This is most likely due to cycling in telomerase activity especially within the root cells [6]. Such a system should preserve a more or less constant condition of tree ring growth, not related to the tree age, creating ideal condition for climate proxy recorder. The precipitation of iron, therefore, should be related to the change of environment.

Climate change can cause rapid changes in microbacterial communities living within the soil, changing the water acidity, and causing the dissolved iron to precipitate and rather than taking parts in various proteins that manage iron equilibrium, iron can be stored within the iron oxide particles as it was shown in mammals [7]. This model creates a convenient test case for magnetic sensing of the tree tissue that may relate to climate changes. In this study, we do not consider atmospheric traffic pollution [8] due to the remote location of the red wood specimen.

## Materials and methods

**Figure 1:**
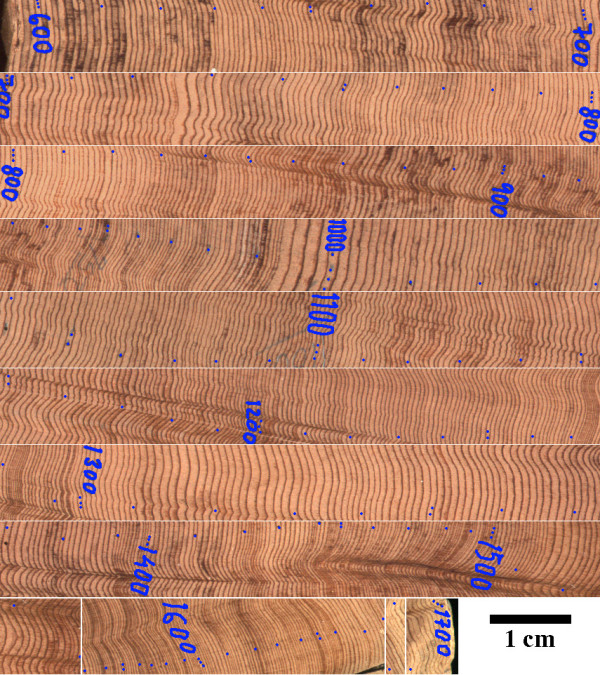
Section of the tree sample that was used for magnetic measurements and tree ring density. Blue numbers indicate a year (AD) when the tree ring was created. Blue dots (pinpricks) help orientation in respect to individual tree ring ages. Each one dot is the 10^th ^year, two vertical dots are the 50^th ^year, three vertical dots are 100^th ^year, and four vertical dots are the 1000^th ^year.

A sample of the Sequoia sempervirens (m26 NE3–NE5) was obtained from professor Malcom Hughes on December 17, 1999, in the Laboratory of Tree-ring research, University of Arizona. The tree sample was collected in Mountain Home State Forest, California (SE of Fresno), and dated by Rex K. Adams. The specimen was cross-dated for the time interval between 950–1450 years. The rest of the years, estimated based on Figure 1, is not cross-dated and may be associated with some errors (+/-5 years). In Figure 1, one pinprick (blue dot) indicates the 10th year, two pinpricks in a vertical alignment indicate the 50th year, three pinpricks in a vertical alignment indicate the 100th year, and four pinpricks in a vertical alignment indicate the 1000th year.

Workers with wood know that getting a magnetically uncontaminated sample is not trivial. In order to obtain pristine samples for magnetic measurements, extra care was applied. Samples were cut by handheld non-magnetic saw in Pruhonice Paleomagnetic Laboratory (PPL), Czech Republic. Wood was collected in year 1998 and stored for one year in Laboratory of Tree Ring Research, Arizona. Within four months after receiving these samples from Laboratory of Tree Ring Research, samples were cut and measured both at GSFC/NASA and PPL. During this time samples were kept in a dry box at GSFC and in relatively dry storage facility of PPL to avoid moisture exposure. During the process of measurement all parts of the sample holder were cleaned with ethyl alcohol and distilled water to insure absence of magnetic contamination.

**Figure 2:**
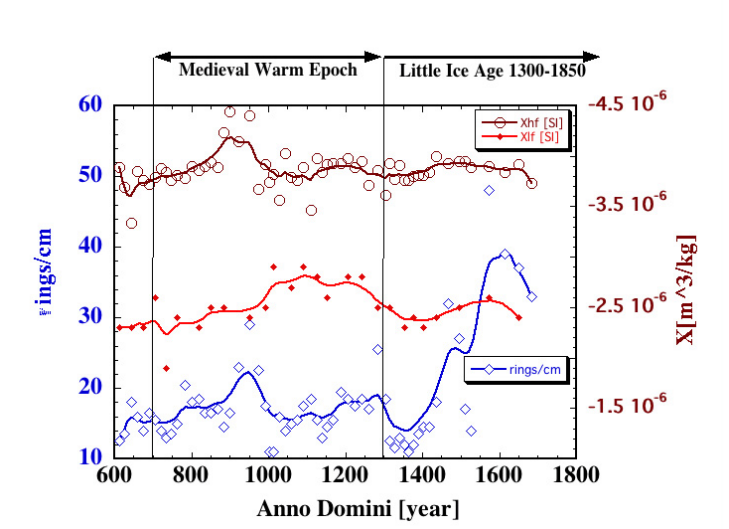
Tree ring density (signal-to-noise ratio ~15/1) and both high- and low-field magnetic susceptibility (susceptibility record has signal-to-noise ratio ~12/1 based on repetitive measurements) are plotted as a function of age. Top of the diagram shows intervals for the Medieval Warm Epoch period [11] and for Little Ice Age [12]. The data are approximated with Stineman function. The output of this function then has a geometric weight applied to the current point and ± 10% of the data range, to arrive at the smoothed curve. This measurement was done at GSFC/NASA.

The NASA specimen was cut into a rod, about 600 mm long, with 100 mm^2 ^cross-section. A sharp scalpel was used to dissect the wooden rod into cubical samples (size ~1 cm). Tree rings were counted within each cube to obtain tree ring density (Figure 2). We estimated that for every 15 tree rings we may have missed or added an extra ring. This allows obtaining a signal-to-noise ratio value of 15/1. These cubes were used to obtain high-field magnetic susceptibility (Figure 2) at GSFC. Low-field magnetic susceptibilities were obtained from the sister samples sent to PPL along with measurements of Natural Remanent Magnetization (NRM), Saturation Isothermal Remanent Magnetization (SIRM), and thermal/alternating field magnetic stability.

High-field magnetic susceptibilities (Figure 2) were obtained at GSFC/NASA from the magnetization change between 1 and 2 Tesla field inside the Vibrating Sample Magnetometer (VSM Model 7300 by Lake Shore, 10 times averaging). The signal-to-noise ratio of this value was estimated to be 12/1 based on repetitive measurements of several samples. Both magnetic slope data and ring density data are approximated using a Stineman function. The output of this function then has a geometric weight applied to the current point and ± 10% of the data range, to arrive at the smoothed curve. Each time the empty holder was measured for the final sample correction. Figure 2 contains low-field magnetic susceptibility values from the sister samples measured in PPL using KLY-2 Kappabridge [9] (frequency 920 Hz, field intensity 300 A/m).

**Figure 3:**
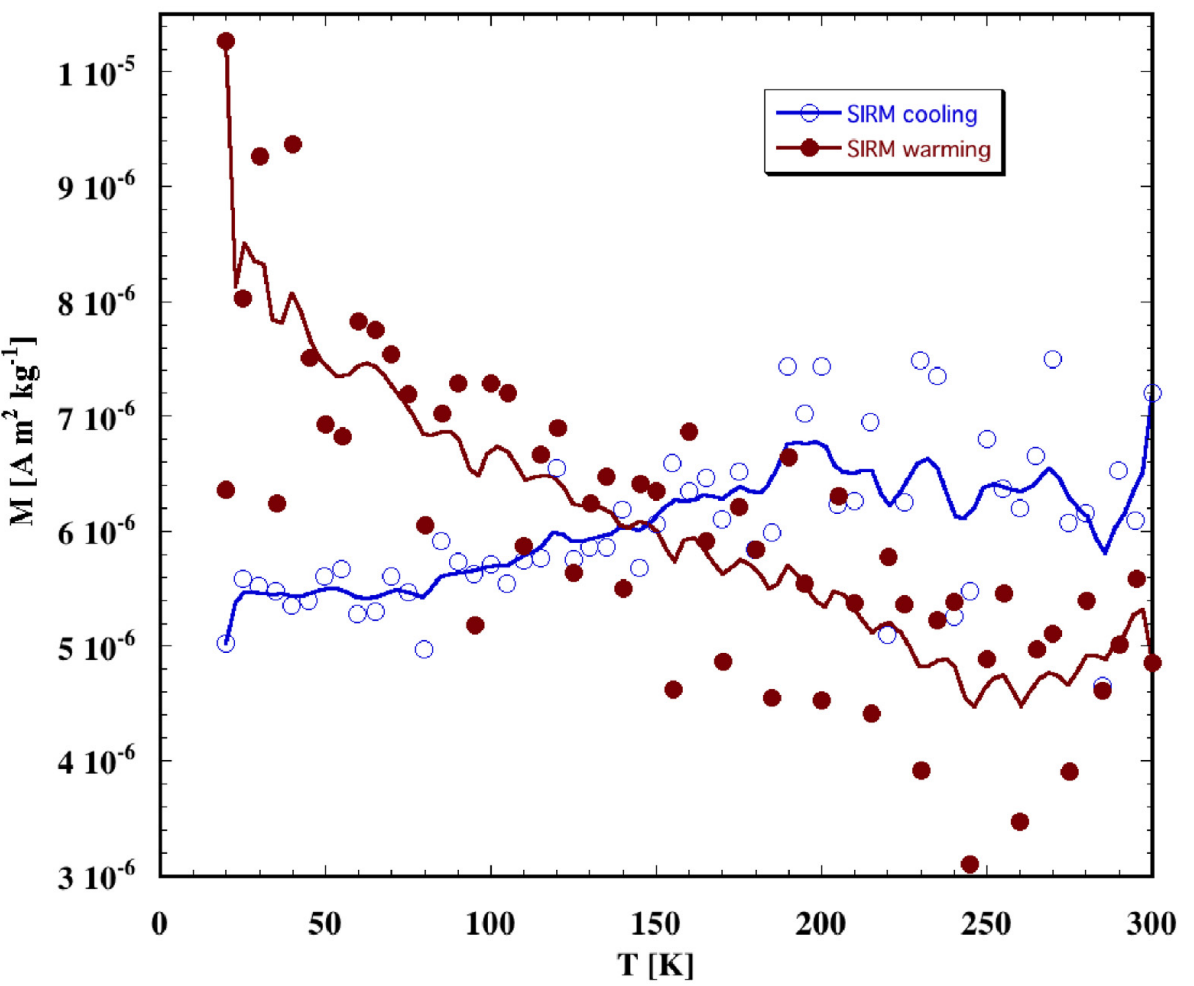
The magnetic remanence is shown as function of temperature during a cryogenic warming (SIRM given at 20 K) and subsequent cryogenic cooling (SIRM given at 300 K). The signal to noise ratio was 3/1. The data are approximated with Stineman function. The output of this function then has a geometric weight applied to the current point and ± 10% of the data range, to arrive at the smoothed curve. Data were taken at MPMS, Institute for Rock Magnetism, Minnesota.

Low temperature magnetic data were obtained at the Institute for Rock Magnetism, University of Minnesota. Small sample (5 mm × 5 mm × 10 mm) from the section near 650 years was cut, using a knife, to fit inside the plastic straw, the holder for Quantum Design MPMS cryogenic magnetometer. The sample was brought to 20 K, acquired saturation remanence in field of 5T and warmed up to 300 K. At 300 K the sample was again given saturation remanence and brought back to 20 K (see Figure 3).

**Figure 4:**
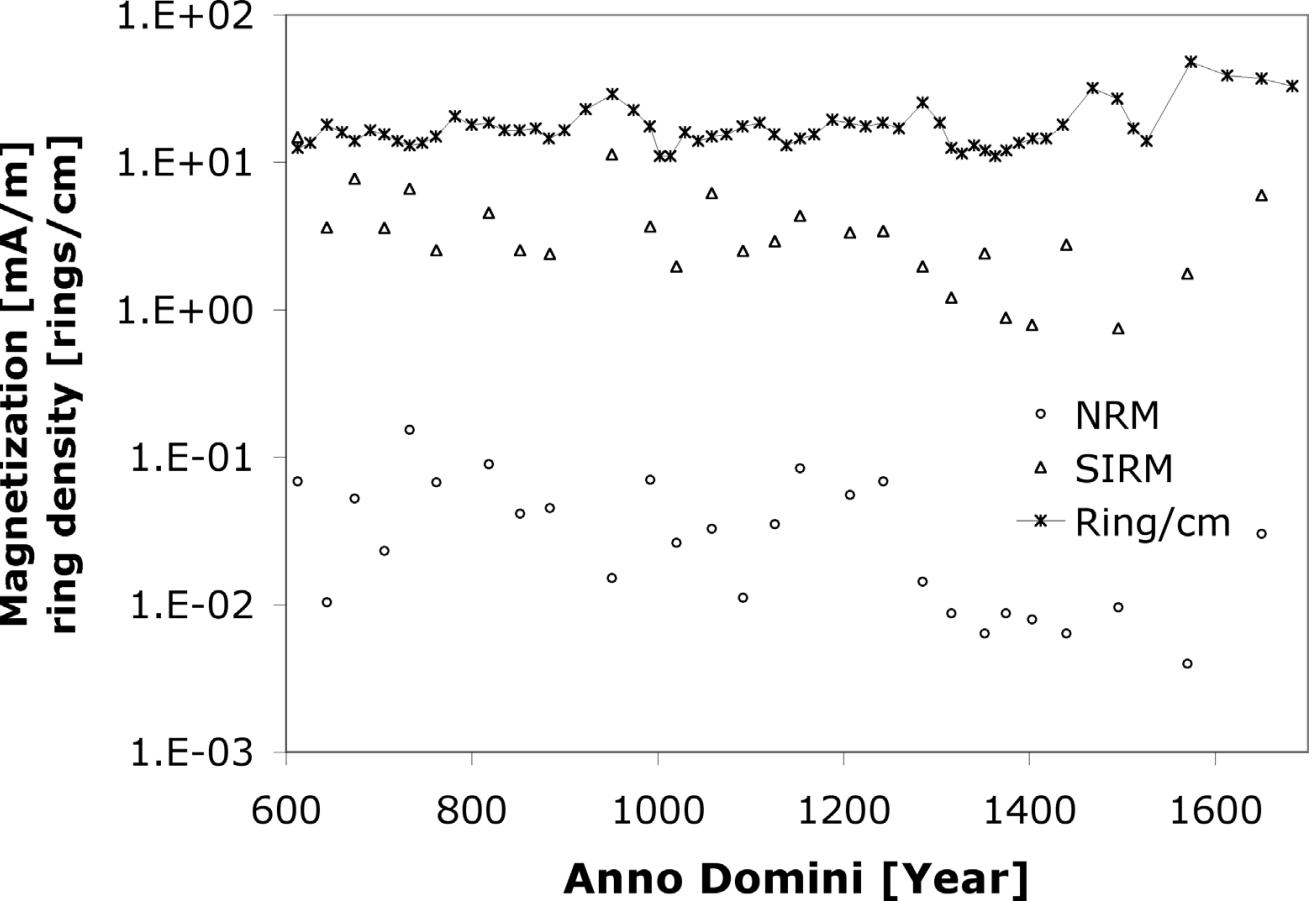
Ring density data are compared with Natural remanent magnetization (NRM, noise limit is 0.0024 mA/m on JR-5A spinner) and Saturation Remanent Magnetization (SIRM) for samples of the wood section sent to Pruhonice Paleomagnetic Laboratory, Czech Republic.

**Figure 5:**
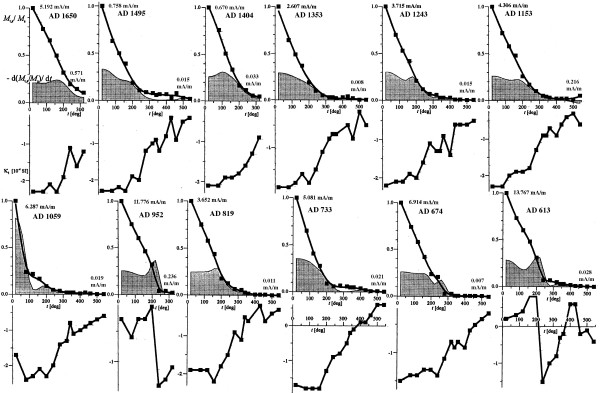
Thermal demagnetization of Saturation Isothermal Remanent Magnetization of selected samples, indicated by age, normalized by the magnetic remanence at room temperature (M_t, s_/M_s_). Derivative is based on the smoothed trend of thermal demagnetization. Magnetic susceptibility after each heating step is shown below remanence plots sharing the temperature axis. The remanence data are approximated with Stineman function. The output of this function then has a geometric weight applied to the current point and ± 10% of the data range, to arrive at the smoothed curve. Data are taken at Pruhonice Paleomagnetic Laboratory (noise limit on JR-5A spinner is 0.0024 mA/m).

**Figure 6:**
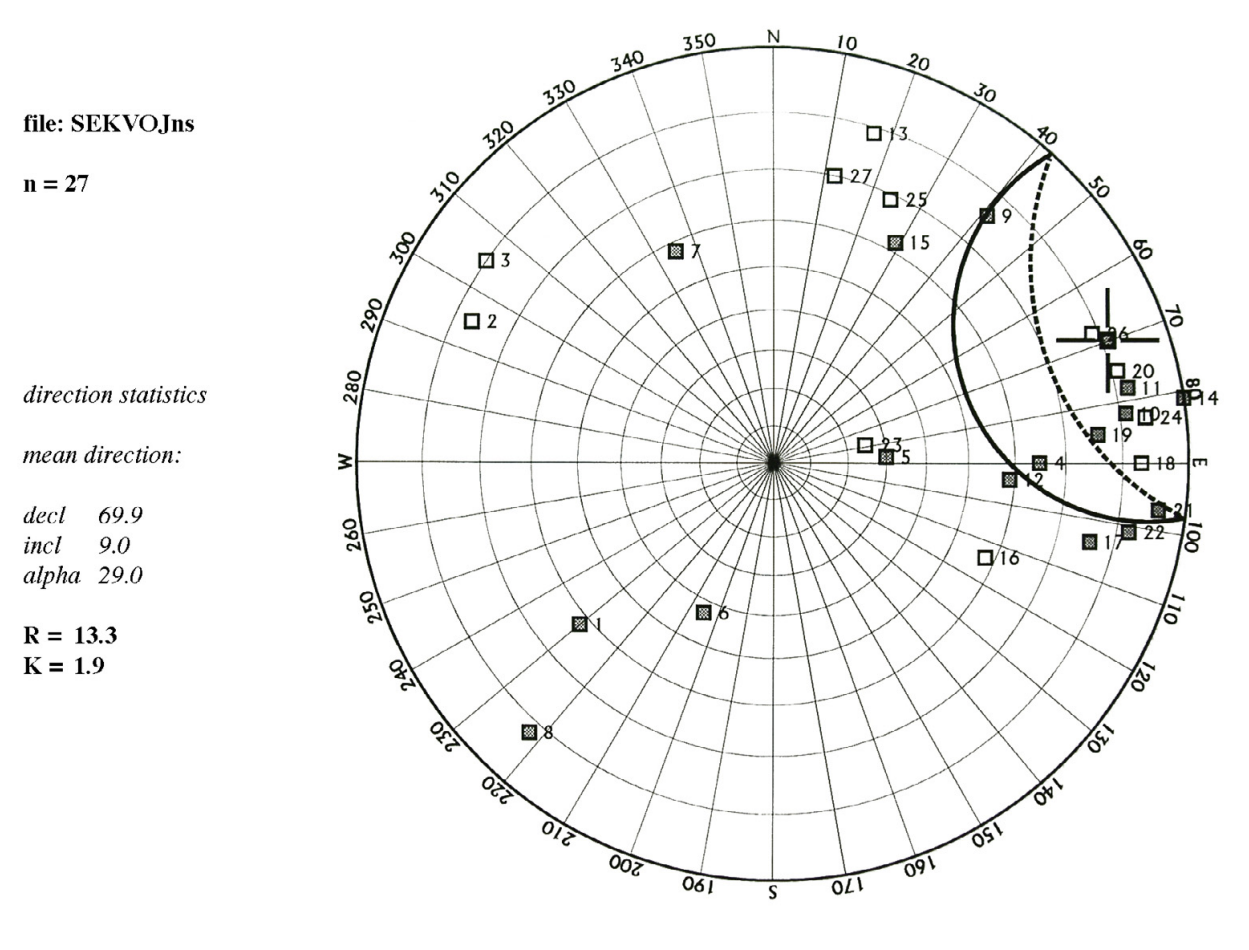
Directional consistency of the sequoia samples measured in Pruhonice Paleomagnetic Laboratory. Solid symbols indicate positive inclination, and empty symbols negative inclination of natural remanent magnetization. Numbers denote relate to the following approximate ages: 1 = year 1650, 2 = year 1613, 3 = year 1495, 4 = year 1436, 5 = year 1389, 6 = year 1376, 7 = year 1352, 8 = year 1316, 9 = year 1285, 10 = year 1242, 11 = year 1224, 12 = year 1153, 13 = year 1138, 14 = year 1110, 15 = year 1059, 16 = year 1030, 17 = year 1003, 18 = year 974, 19 = year 899, 20 = year 851, 21 = year 818, 22 = year 782, 23 = year 747, 24 = year 720, 25 = year 691, 26 = year 660, 27 = year 613.

The PPL obtained NRM and SIRM from sister samples (Figure 4) using JR-5A Spinner Magnetometer (measuring range from 2.4 e-3 to 1.6 e+3 mA/m). SIRM was acquired in an electromagnet (Polytechnik, Germany) using a direct magnetic field to the state of saturation at the maximum field intensity of 1 T. SIRM of 12 selected samples were partially thermally demagnetized (Figure 5) with MAVACS (Magnetic Vacuum Control System). This instrument creates a magnetic vacuum less than 1 nT [10]. Each demagnetization step was followed by measurement of magnetic susceptibility (frequency 875 Hz, field intensity 300 A/m) with KLY-2 Kappabridge [9]. Changes in magnetic susceptibility indicate chemical changes within the sample during the heating (see Figure 5). Five samples with SIRM from the older section of the tree (600–1000 years A.D.) were demagnetized by alternating field up to 0.1 T with instrument Schonstedt GSD-1 demagnetizer and subsequently stepwise magnetized by field up to 0.5 T. All magnetic remanence measurements were done such that samples were kept oriented in respect to each other. NRM magnetizations from all samples stayed within +/- 30 degrees (see PPL data set, Figure 6).

## Results and discussion

Tree ring density was counted within individual specimens to obtain the tree ring density shown in Figures 2 and 4. A more precise variation in tree ring density can be obtained by direct measurements of the tree ring size of the image shown in Figure 1. However, the specific tree ring density should be specifically related to samples used in magnetic experiments.

Tree ring density (TRD) shown in Figure 2 indicates several episodes where not much wood material was added, suggesting much slower growth. First episode is between 900 and 1000 A.D. Second is less pronounced between 1200 and 1300 A.D. The most dramatic increase in TRD outlines the most recent section of the wood dated between 1400 and 1700 A.D. The relative TRD indicates rapid cellular growth when the climate was likely warmer and wetter. The most pronounced episode based on TRD is between 1300 and 1400 A.D, just near the end of the Medieval Warm Epoch [11] and start of the Little Ice Age in North American Coast Mountains [12] (see Figure 2). Other periods where the climate favored the cellular proliferation are between 650 and 750 A.D. and between 1000 and 1150 A.D. Interestingly, these outlined episodes of contrasting cellular proliferation weakly correlate with high field diamagnetic susceptibility (the denser the tree rings the more diamagnetic material is present with linear correlation coefficient R = 0.14, see Figure 2). When cellular growth is suppressed the diamagnetic signature is intensified. The material used for cellular growth may contain more carbon atoms, therefore raising the diamagnetic signature. For the period between 1400 and 1700 A.D., the diamagnetic enhancement is not as dramatic as it is for the tree ring density. This may be related to the proximity of the actual terminus of the tree that contained the living tissue at the time of the tree death.

The data suggest that the larger the amount of magnetic carriers the larger the value of high-field diamagnetic slope. Diamagnetic and paramagnetic or superparamagnetic carriers can cause these slope variations. Since a significant part of the magnetic signature appears to be superparamagnetic, our data suggest that less dense wood contains more paramagnetic and superparamagnetic material irrespective to the amount of magnetic remanence carriers (presumably saturated).

The cryogenic experiments on MPMS suggest continuous unblocking of the remanence on warming due to the presence of superparamagnetic grains (Figure 3). There is no indication of the Verwey transition. Cooling of Room temperature SIRM resulted in no significant change in remanence magnetization (Figure 3). We did not attempt to image magnetic carrier as it is likely to be only visible by transmission electron microscopy and we do not have such facility currently available.

Individual magnetic remanence records (NRM and SIRM) were too noisy to infer any climatic relations (Figure 4) between magnetization and tree ring. Magnetization amplitudes (SIRM) were consistent with the remanence measured at different temperatures with Quantum design MPMS instrument, where the SIRM at room temperature corrected for density (500 kg/m^3^) is near 3 mA/m (see Figure 3). In summary, NRM and SIRM records shown in Figure 4 indicate that there is no significant correlation between magnetizations and tree ring density.

### Magnetic efficiency

The precipitation of the NRM carriers may be completely unrelated to the paleoclimate and this is supported by results in Figure 4. Therefore, we decided to test the wood samples for magnetization efficiency (NRM/SIRM ratio) that often reveals more detailed magnetic remanence characteristics in terms of the thermal magnetization (TRM) component. Note that the SIRM is about 75 times larger than NRM (Figure 4). This is similar to the efficiencies where NRM remanence of thermal origin [13,14]. TRM is when material is heated above the blocking temperature of the residing magnetic carriers and subsequently cooled down in ambient magnetic field. Chemical remanence magnetization (CRM) has similar physics of magnetic acquisition. Magnetic grains grow into the larger volumes during the convenient chemical conditions in ambient temperature. Once the particles' volume reaches single domain magnetic state the CRM component is blocked and therefore sample acquires CRM component.

**Figure 7:**
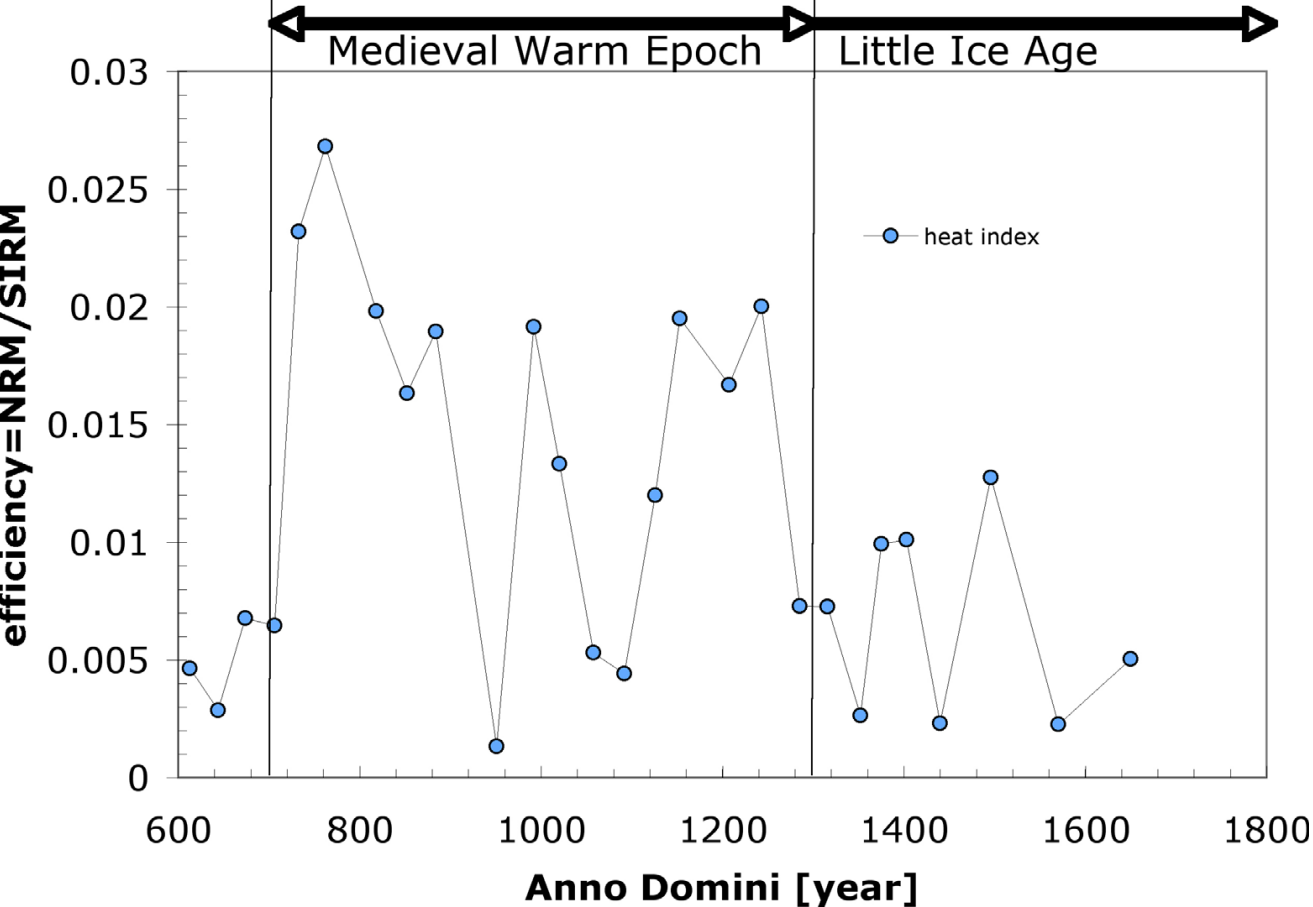
Efficiency of magnetization that approximate "heat index" is plotted as a function of age for samples of *Sequoia sempervirens*. Top of the diagram shows intervals for the Medieval Warm Epoch period [11] and for Little Ice Age [12].

It is conceivable that magnetic grains warm above their blocking temperatures during heat anomaly events (fire/drought) and cool down to block the thermal remanent component. The relative magnetic signature fluctuation is noisier for NRM data set than SIRM (Figure 4) and this possibly relates to a demagnetization event that may have influenced the original remanence after it was acquired in nature. We infer several NRM components. The first component is chemical remanence (CRM) because we assume that the magnetic minerals had to be formed from within the tree tissue below the blocking temperatures of the remanence carriers. Under ambient temperature a significant fraction of the grains is likely to be in the superparamagnetic (SP) domain state with grain size <30 nm. Therefore, the second NRM component can be partial thermoremanent magnetization with blocking temperatures that span across the ambient temperatures. Some evidence supporting this claim is in Figure 5, where the SIRM is rapidly declining during the partial thermal demagnetization in ambient air. The rapid decay is due to thermal unblocking of the remanence. Because of the steepness of the slope, it may be conceivable that daily heating due to weather may continuously block and unblock parts of the remanence. When anomalous heating has been recorded it is sealed from the future thermal fluctuations by additional wood growth. We must say, however, that prolonged storage of these samples containing SP grains could also generate a third component of viscous remanent magnetization (VRM) adding to the overall value of NRM. For example assuming that the subset of these samples had large amount of SP grains, they would be prone to viscous re-magnetization, most likely randomizing the original signature. If this would be the case we would observe significant directional deviation of NRM within the sample set. In Figure 6, we plot directions of the PPL data set and show that only few samples (6 out of 27) may have considerable directional change, and indeed, the angular deviation of the largest outliers is associated with the low sample efficiencies in Figure 7.

Samples labeled as AD 952 and AD 613 in Figure 5 show anomalous behavior, both in susceptibility (positive on start) and in shape of the demagnetizing curve (bell like shape) suggesting a presence of very fine magnetic grains on the surface of these samples that quickly oxidizes and or gets removed during the sample handeling when heated over 200°C. Note that these two samples have a much higher initial SIRM intensity (12 mA/m and 14 mA/m respectively). Therefore, heating causes a rapid susceptibility swing to negative values (ferromagnetic part is removed) as well as bell shape decay curves (samples AD 952 and AD 613 in Figure 5). Most of other samples have smaller initial magnetization and do not show such anomalous behavior. Heating is associated with overall reduction and stabilizing iron rich complexes into oxide minerals. New ferromagnetic material is evidenced by removal of diamagnetic component in susceptibility plots (Figure 5). The true nature of the susceptibility and mineralogy of the remanence carriers is speculative, however. We suggest maghemite due to low thermal magnetic stability along with the absence of Verwey transition (Figures 3 and 5).

**Figure 8:**
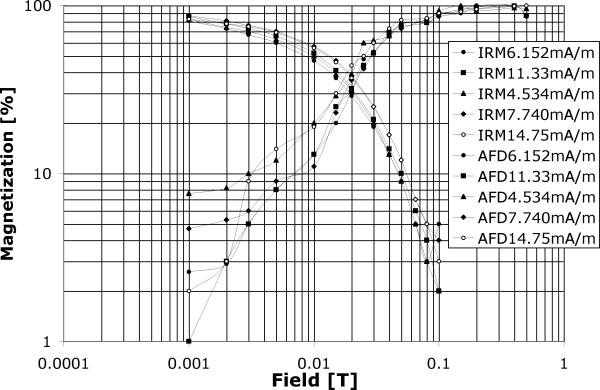
Alternating field demagnetization (AFD) and Isothermal remanence acquisition (IRM – volume normalized) measured on JR-5A spinner magnetometer (noise limit 0.0024 mA/m) at paleomagnetic lab, Pruhonice, Czech Republic. Saturation magnetization is shown in the legend. These 5 samples come from the section with tree rings in the range of 600–1100 years (AD). From top down they correspond to the following years; 1059, 974, 818, 691, and 613.

Demagnetization by alternating demagnetizing field along with IRM acquisition (Figure 8) shows uniform behavior across 5 samples (AD 1059, AD 974, AD 818, AD 691, and AD 613). Sample AD 613 has initial remanence of 14.75 mA/m. Such high SIRM value may be related to contamination similar to the one shown for samples AD 952 and AD 613 during the thermal heating. This sample showed slightly more positive interaction compared with other samples, which would be consistent with surface contamination where the contaminants are likely clustered together rather then evenly distributed over the surface. Medium demagnetizing field is about 0.01 T suggesting higher coercivity and therefore overall remanent stability. Such behavior indicates that viscous overprint may be not relevant for these samples.

The rapid decrease of remanence due to slight heating reveals that there may be thermal history recorded within the wood samples. A remanence record can be characterized by plotting efficiency (Figure 7) of the natural remanent magnetization (NRM/SIRM). In regular conditions the efficiency of Thermal remanent magnetization (TRM) component of NRM should be very close to 1–2% [13,15]. However, this value often drops well below 0.01 in Figure 7. The efficiency of CRM component of NRM is likely to be less than efficiency of TRM component. This is because, if superparamagnetic (SP) grains are present, the saturation magnetizations of SP grains that do not normally contribute to CRM, cause SP grains to interact among each other and contribute to the overall saturation remanence. If SP grains are not saturated (CRM), they do not sense each other and therefore do not contribute to the overall CRM signature. For this reason, the CRM component of efficiency in a sample containing SP grains must be lower.

We propose that there are sections of the wood that have been heated in the past by climate variation (including fire), inducing the partial TRM component into the wood. Plotting the efficiency in Figure 7 depicts wood sections that have been affected by heat and identify these sections as with larger efficiency. Thus the samples with efficiency exceeding 0.01 would be likely to undergo some thermal event, strengthening its NRM intensity by mild heating (e.g. forest fire or climate change). This proposed variation is consistent with the rapid unblocking of remanence seen in Figure 5. Our identification of the highly thermally dependent magnetic signature opens the possibility that trees may contain pTRM record. Since trees are generally only few thousands years old (e.g. sequoias) the time may be short enough for a tree to record stable thermal history. Our data outline this possibility for thermal record preservation and suggests more general testing of such hypothesis.

We note that the size of the samples is rather large compared to the growth rings and leads to aliasing of the magnetic record. Using the average tree ring density in Figure 3 as 15 rings per centimeter we estimate that NRM magnetic signature per one tree ring as in the range of 10^-3 ^mA/m, which is below the limit of our instruments. However, there are more sensitive instruments being developed by 2 G and quantum design and paleomagnetic signature of the individual tree rings may not be impossible in near future. In this study the aliasing effect may cause some reduction of significant anomalous climatic events happening on scale smaller than ~15 years. We note, however, that our proposed thermal mechanism for partial TRM of the tree samples may also cause some degree of aliasing on the tree ring scale due to thermal flow inwards that would be competing with natural cooling capacity of the tree to maintain lower temperature than ambient.

## Conclusion

Wood material from the *Sequoia sempervirens *contains variable tree ring density indicating the environmental changes and health status during the life span of the tree. The tree ring density correlates weakly with the high field magnetic susceptibility, suggesting accumulation of the diamagnetic material within the zones of high tree ring density. This correlation is less pronounced near the tree perimeter possibly due to proximity of the tree section that was living at the time of the tree death. Correlation with the remanence is absent (Figure 4). Cryogenic measurements suggest continuous unblocking of the remanence during heating and thus lowering the sample intensity in the observed NRM measurements. NRM/SIRM measurements suggest presence of thermal component of the remanence within the trees. NRM tends to fluctuate to larger amplitudes than SIRM. This fluctuation may be due to blocking temperature that is very close to room temperature. Therefore, we attempted to use remanence efficiency to characterize a thermal exposure history of the tree. Such an approach offers a potentially important climate proxy, the record of the peak temperature during the tree ring formations. This assumes that the fluids transporting the nutrients to the tree efficiently cool the interior of the tree and only the very exterior part is exposed to more extreme environmental changes.

The high-field susceptibility variations together with the TRD proxies document in detail the cold oscillation between 900 and 1000 A.D. preceding the Medieval Warm Epoch lasting until the end of 14th century. The proxies obtained from the Sequoia sempervirens magnetic efficiency (Figure 7) were able to record the steep climate cooling triggered by the Little Ice Age after ca 1400 A.D., which is in agreement with reconstructed Northern Hemisphere temperatures [16,17].

This work is mostly an exploratory attempt to see if magnetic analysis of trees may be useful. Our report suggests that there is a signature that may reflect the thermal history and that the tree contains magnetic carriers that recorded ambient field at the time of growth. It may be that in the future, more detailed research of individual tree rings could reveal differences in the magnetic environment at the time of magnetization origin. This could possibly aid to the knowledge of the historical geomagnetic field variation as well as detailed magnetic field fluctuation on yearly bases.
